# Survey on ICP, target CPP and MAP measurement level in patients with severe acute brain injury in different ICUs

**DOI:** 10.1186/cc14524

**Published:** 2015-03-16

**Authors:** M Van Laer, K Deschilder, W Stockman

**Affiliations:** 1 UZ Brussel, Brussels, Belgium; 2 AZ Delta, Roeselare, Belgium

## Introduction

**Figure 1:**
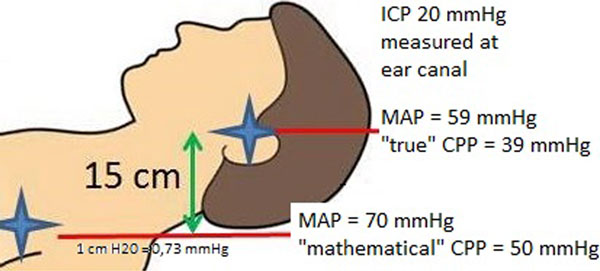


Since most patients with acute brain injury are treated head-up at 30 to 45°, there can be a height difference of up to 15 cm between the heart and the ear canal. This causes a difference between mathematical CPP and true CPP of up to 11 mmHg depending on the zero reference level used and the body length of the patient (Figure 1). We conducted a survey to analyze the current practice on CPP targets and zero reference levels in different ICUs.

## Methods

Neuro-ICU physicians were invited to participate in a survey on ICP and CPP targets and the level of measurement.

## Results

The results of 30 centers are summarized in Table 1. Most centers (83.3%) use head-up elevation of 30 to 45° and consider an ICP of 20 mmHg as the threshold to start treating ICP (80%). More variation is noted in minimal and maximal CPP threshold. All centers measure ICP at the ear canal. Twenty-seven centers (90%) measure MAP at the heart, three centers measure MAP at the ear canal. These three centers use >50, 60 and 65 mmHg as minimal CPP and raise the bed to 30 to 45°. The two centers using minimal CPP >60 mmHg do not apply an upper limit.

**Table 1:** 

ICP threshold (mmHg); *n *(%)	>15; 2 (6.7%)
**>18; 1 (3.3%) **	**>20; 24 (80%) **

**>25; 3 (10%)**	

Minimal CPP (mmHg); *n *(%)	>50; 6 (20%)
>55; 4 (13.3%)	>60; 16 (53.3%)
>65 to 70; 4 (13.3%)	
Maximal CPP (mmHg); *n *(%)	<70 to 75; 8 (26.7%)
<80; 8 (26.7%)	<85; 2 (6.7%)
No limit; 12 (40%)	

## Conclusion

Considering the influence of position on CPP, the variations among centers and the narrow range of CPP thresholds, future studies and guidelines should describe where MAP is measured. Alternatively, we propose a new formula for CPP: true CPP = MAP(heart) - ICP(ear) - height difference (heart to ear canal (cm)) × 0.73.

